# Antimicrobial resistance in patients with odontogenic infections: A systematic scoping review of prospective and experimental studies

**DOI:** 10.4317/jced.59830

**Published:** 2022-10-01

**Authors:** Carlos-M. Ardila, Jader-Alexander Bedoya-García

**Affiliations:** 1 DDS. Periodontist. Ph. D in Epidemiology. Postdoc in Bioethics. Titular Professor. Universidad de Antioquia U de A, Medellín, Colombia; 2 Biomedical Stomatology Research Group, Universidad de Antioquia U de A, Medellín, Colombia; 3 DDS. Periodontist. Professor. Universidad de Antioquia U de A, Medellín, Colombia

## Abstract

**Background:**

Patients with odontogenic infections are commonly prescribed antimicrobials on an experiential base without knowing the precise microorganisms implicated. The aim of this systematic scoping review is to evaluate the prevalence and proportions of antimicrobial-resistant species in patients with odontogenic infections.

**Material and Methods:**

A systematic scoping review of scientific evidence was accomplished involving different databases.

**Results:**

Eight randomized clinical trials and 13 prospective observational studies were included. These investigations analyzed 1506 patients. The species that showed higher levels of resistance included aerobic and facultative anaerobe such as *Staphylococcus aureus, Streptococcus viridans, Klebsiella pneumoniae, Streptococcus milleri, Enterococcus spp., Pseudomonas aeruginosa, Proteus mirabilis*, and *Staphylococcus coagulases-negative*. In obligate anaerobes sampled were Peptostreptococcos spp., Bacteroides spp., and Prevotella spp. Staphylococcus showed resistance to ampicillin, piperacillin, clindamycin, amoxicillin, metronidazole, and penicillin. Streptococcus had resistance to metronidazole, clindamycin, doxycycline, penicillin, and amoxicillin. Peptostreptococcus spp. presented resistance to penicillin, amoxicillin, erythromycin, and cefalexin. Gram-negative microorganisms had resistance to tetracycline, ciprofloxacin, azithromycin, amoxicillin, erythromycin, and penicillin. Bacteroides spp. exhibited resistance to penicillin, erythromycin, and gentamicin. Prevotella spp. showed resistance to penicillin, amoxicillin, erythromycin, clindamycin, levofloxacin, and imipenem. Finally, Klebsiella spp. displayed resistance to ampicillin, amoxicillin, moxifloxacin, and cefalexin. Interestingly, one clinical trial showed that after therapy there was a reduction in sensitivity of 18% for azithromycin and 26% for spiramycin.

**Conclusions:**

Most of the microorganisms had resistance to diverse groups of antimicrobials. Suitable antimicrobials must be prescribed founded on the microbial samples, culture susceptibility, and clinical progression of the odontogenic infection. Furthermore, it was observed high levels of resistance to antimicrobials that have been used in local and systemic therapy of oral cavity infections. A preponderance of anaerobic microorganisms over aerobic ones was observed.

** Key words:**Antibiotic resistance, odontogenic infections, efficacy, microorganisms, scoping review.

## Introduction

Odontogenic infection is the most commonly appearing infection in the orofacial area. These infections comprise from periapical abscesses to mild and profound infections in the neck and are frequently caused by periodontitis and dental caries as well as pericoronitis and complications during dental procedures (1).

It has been recognized that the treatment of odontogenic orofacial and neck infections is mainly oriented to the clinical alleviation of suppuration. Nonetheless, antimicrobials adjunct to that therapy is relevant, particularly when there is systemic compromise (2). The empirical choice of appropriate antimicrobials for the management of these infections is supported by their clinical efficacy, low prices, few adverse events, and good availability (3).

This empirical management has generated complications related to the use of antimicrobials, an issue that in turn has allowed investigating of regular prescription practices by dentists (4,5). The selection of antimicrobial for the treatment of odontogenic infections preferably requires the performance of a microbial culture to carry out susceptibility tests. Nevertheless, it has been indicated that 46% of dentists from different countries disregarded this conduct before the recommendation of antimicrobials, albeit 83% of the total clinicians interrogated were conscious of the growth in antimicrobial resistance (5). Therefore, patients with these odontogenic infections are commonly prescribed antimicrobials on an experiential base without knowing the precise microorganisms implicated. This antimicrobial management could or could not generate satisfactory effects due to diverse reasons such as bacterial specificity and antimicrobial resistance (6).

On the other hand, geographical differentiation, the occurrence of resistant microorganisms, and native antibacterial prescribing policies generate variability in the antimicrobial profile of pathogens between communities (7).

Since the development of antimicrobial resistance during antimicrobial management in dental practice is a matter of concern, it is relevant to carry out a scoping review that allows for evaluating the antimicrobial resistance patterns by phenotypic identification of the microorganisms most commonly isolated from odontogenic infections. To achieve this objective, it was proposed to answer some questions related to antimicrobial resistance, in terms of the prevalence and proportions of antimicrobial-resistant species in odontogenic infections. Furthermore, the antimicrobials to which the odontogenic pathogens present resistance were also investigated.

## Material and Methods

This review of prospective and experimental studies in humans was carried out considering the PRISMA (Preferred Reporting Items for Systematic Reviews and Meta-analyses) extension for scoping reviews (8). The scoping structure involved different databases such as PubMed/MEDLINE, SCOPUS, SCIELO, and LILACS, including the gray literature. MeSH terms and keywords were used to investigate publications in all languages until March 2022, integrating the terms odontogenic, infections, antibacterial drug resistance, dental infection, antibiotic resistance, antibiotics, alveolar abscess, dentoalveolar abscesses, antibacterial susceptibility breakpoint determination, bacterial sensitivity tests, and prospective and experimental studies. Then, a searching process was implemented to explore databases using Boolean operators (AND, OR): “odontogenic” OR “infections” OR “antibacterial drug resistance” OR “dental infections” OR “antibiotic resistance” OR “antibiotics” AND “dentoalveolar abscesses” AND “antibiotic resistance” OR “antimicrobials” OR “alveolar abscess” OR “microorganisms” OR “antibacterial susceptibility breakpoint determination”, OR “bacterial sensitivity tests”.

-Resources selection

Only prospective and experimental studies involving persons diagnosed at the beginning of the study with moderate to severe orofacial/dentoalveolar infection of odontogenic origin, and studies containing phenotypic analysis and antimicrobial susceptibility results were selected. Furthermore, lactating and pregnant women, patients in whom it was not feasible to acquire a proper pus sample, or if systemic antimicrobial was not necessary, or presenting a coexisting systemic illness, were not contemplated for this review. Duplicate publications and analyses applied to animals were also not incorporated.

-Questions

This scoping review aims to answer the following questions: What is the prevalence of antimicrobial-resistant species in patients with odontogenic infections? What is the proportion of antimicrobial-resistant species in patients with odontogenic infections? To which antibiotics did the microorganisms show resistance?

-Review process

Both investigators assessed the titles and abstracts and chose prospective and experimental studies to consider the full text for probable suitability. In case of discrepancy among authors, research eligibility was defined by agreement. The Kappa test was implemented to calculate the score of agreement among researchers (>85).

-Data collection

A Table was considered to include the most pertinent information from the chosen reports. This procedure was completed individually by each of the investigators. Successively, the records were compared. Documented information contained authors’ names, date of publication, amount of patients and quantity of isolates assessed, the occurrence of antimicrobial-resistant bacteria, the percentage of antimicrobial-resistant microorganisms, and antimicrobials in which resistance was observed.

-Risk of Bias

Both authors of this scoping review, independently assessed the methodological quality of the included investigations, using a previously described instrument (9). The instrument contains 16 conditions. A value from 0 to 3 is given to each criterion (0=it does not provide the level of detail needed to generate a decision for a criterion; 1=slightly provided; 2=moderately provided; 3=completely provided). The sum of these criteria gives a total result for the body of evidence, stated as a proportion of the maximum probable score.

## Results

**Figure 1:**
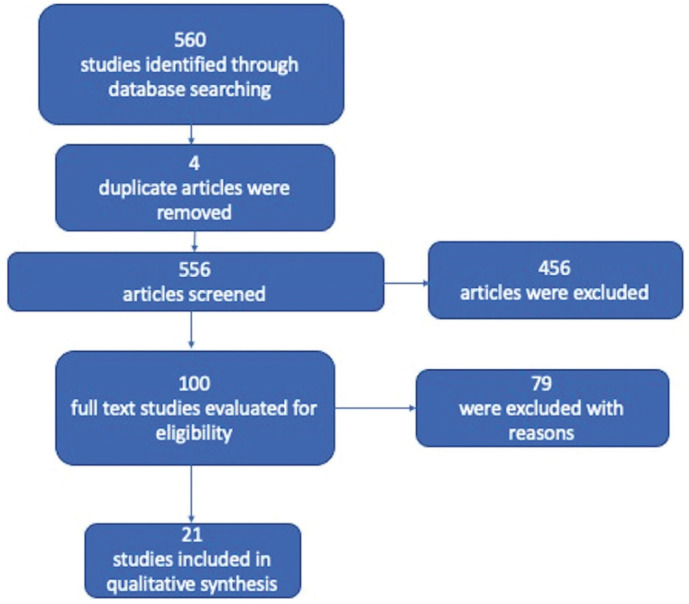
Flowchart of the studies selection method.

The electronic exploration conceded 560 investigations. After evaluating the titles and abstracts, 79 studies were eliminated for their unimportance, and 4 duplicate publications were also ignored. Reading the full text occasioned the omission of 456 additional investigations because they did not meet some selection criteria. Finally, 8 randomized clinical trials (RCTs) (10-17) and 13 (18-30) prospective observational studies were included in this scoping review (Fig. 1).

The features of the incorporated studies are shown in Table 1- Table 1cont.-3. These researches were published between 1987 (17) and 2021 (24). These investigations assessed 1506 participants with a minimum sample of 21(10) patients and a maximum of 142 (26).

**Table 1:**
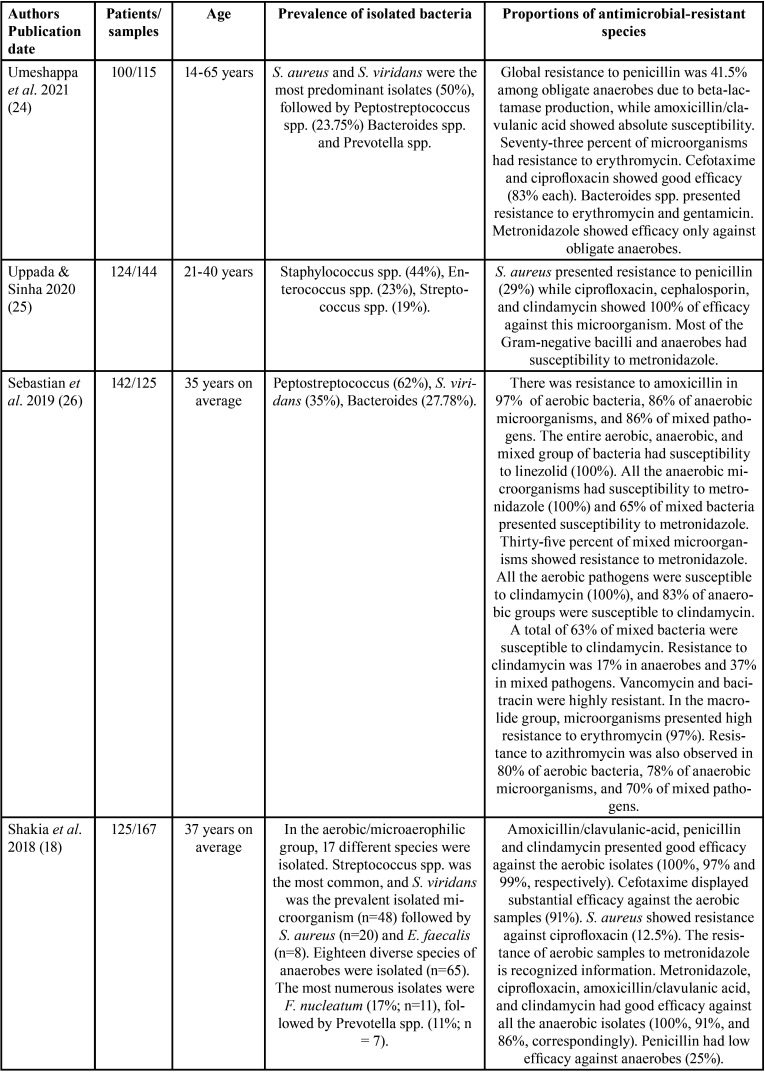
Features of the studies evaluated.

**Table 1 cont.**
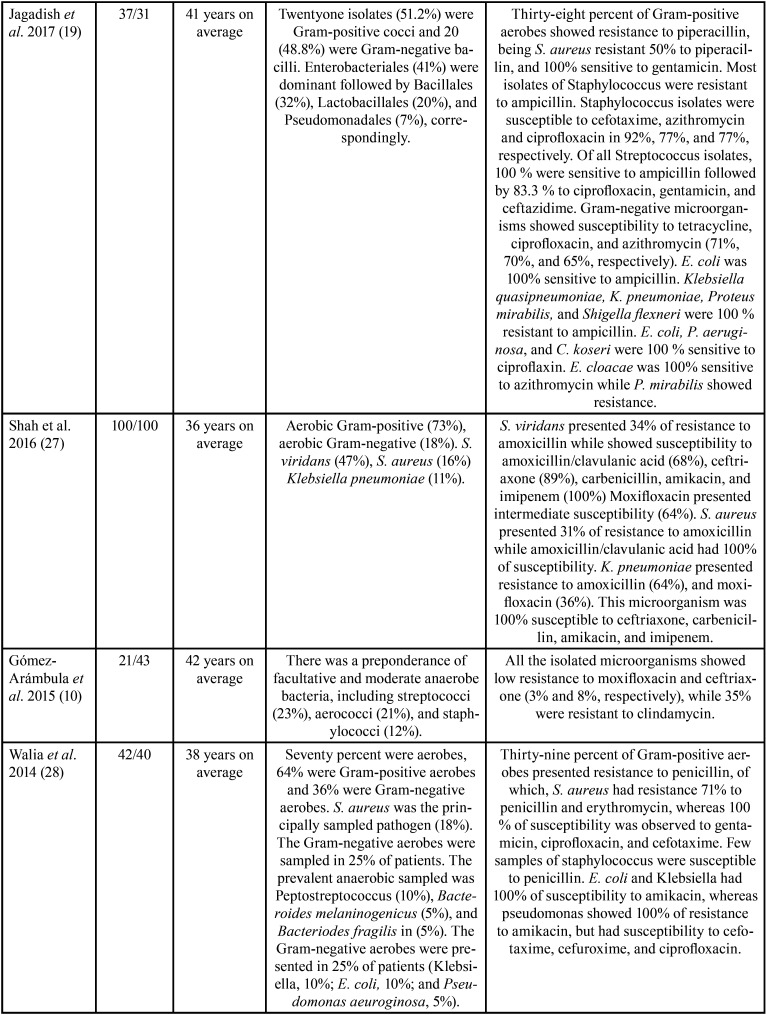
Features of the studies evaluated.

**Table 1 cont.-1:**
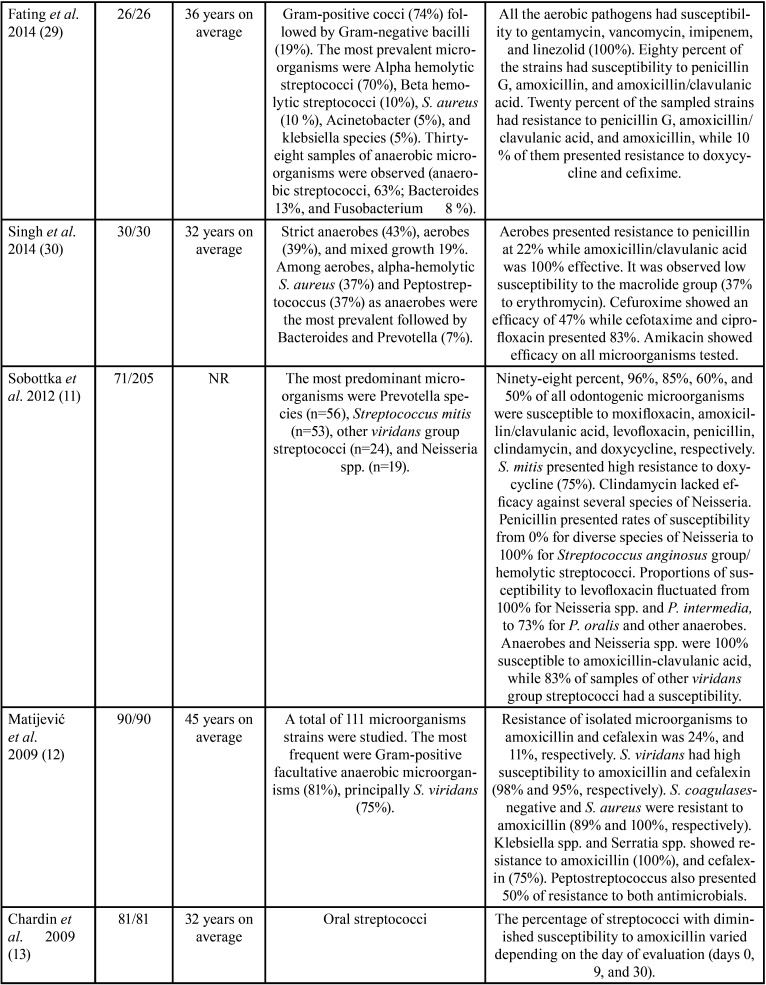
Features of the studies evaluated.

**Table 1 cont.-2:**
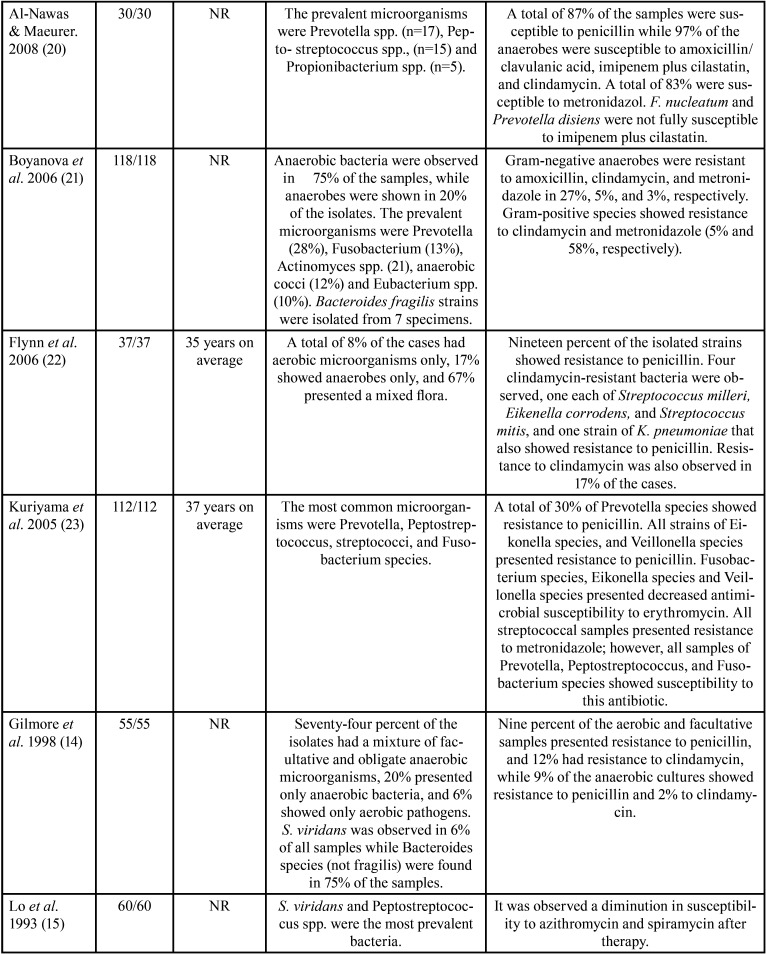
Features of the studies evaluated.

**Table 1 cont.-3:**
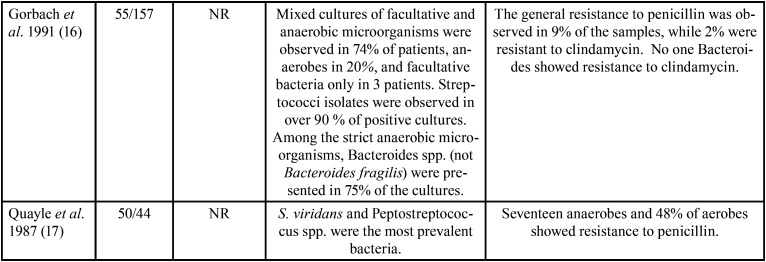
Features of the studies evaluated.

Antibiotic resistance to a wide variety of antimicrobials was explored, including cephalosporins, metronidazole, penicillin, amoxicillin, amoxicillin-clavulanic acid, tetracycline, doxycycline, clindamycin, ampicillin, ciprofloxacin, gentamycin, erythromycin, azithromycin, imipenem, spiramycin, linezolid, vancomycin, bacitracin, amikacin, piperacillin, moxifloxacin, and levofloxacin. However, the most studied antibiotics were penicillin, clindamycin, metronidazole, amoxicillin and, amoxicillin/clavulanic acid.

On the other hand, concerning the prevalence of antimicrobial-resistant species, it was observed that a great variety of microorganisms were isolated (Table 1); nevertheless, the species that showed higher levels of resistance included Staphylococcus, *Streptococcus spp*., Peptostreptococcus spp., Prevotella spp., and Bacteroides spp. Among aerobic and facultative anaerobe prevailed, *Staphylococcus aureus* (12,18,19,25,28), *Streptococcus viridans* (15,17,26,27), *Klebsiella pneumoniae* (12,19,22,27), *Streptococcus milleri* (22), Enterococcus spp. (25), *Pseudomonas aeruginosa* (28), *Proteus mirabilis* (19), and *Staphylococcus coagulases-negative* (12). In obligate anaerobes sampled were Peptostreptococcos spp. (12,15,17,18,20,21,23,24,26,30), Bacteroides spp. (12,14,18,21,24,26,28-30), and Prevotella spp. (11,18,20,21,23,26,30).

All the investigations implemented different protocols for the identification of the microorganisms studied. Nonetheless, most researchers used the disk diffusion protocol to check susceptibility to antimicrobials and interpreted following recognized guides.

Table 1 also details the different proportions of antimicrobial-resistant species. The studies found that Staphylococcus showed resistance to ampicillin (19), piperacillin (19), clindamycin (10), amoxicillin (12), metronidazole (18,21), and penicillin (17,24,25,28-30). Streptococcus had resistance to metronidazole (23), clindamycin (10), doxycycline (11), penicillin (28,29), and amoxicillin (26,27,31). Peptostreptococcus spp. presented resistance to penicillin (24,30), amoxicillin (12,26), erythromycin (30), and cefalexin (12,30). Prevotella spp. showed resistance to penicillin (18,23), amoxicillin (21), erythromycin (23), clindamycin (26), levofloxacin (11), and imipenem (20). Gram-negative microorganisms had resistance to tetracycline, ciprofloxacin, azithromycin (19,26), amoxicillin (21,26), erythromycin (23,24), and penicillin (18,23,24). Bacteroides spp. displayed resistance to penicillin (24), erythromycins (24,30), and gentamicin (24). Finally, Klebsiella spp. exhibited resistance to ampicillin (19), amoxicillin (12,27), moxifloxacin (27), and cefalexin (12). Interestingly, in one RCT, at baseline 75% of *S. viridans* and Peptostreptococcus spp. were susceptible to azithromycin and 63% to spiramycin. However, after therapy, 57% had susceptibility to azithromycin and 37% to spiramycin, with a reduction in sensitivity of 18% for azithromycin and 26% for spiramycin (15). Similarly, in another RCT, the percentage of streptococci with diminished susceptibility to amoxicillin ranged from 1.3 % of the total streptococci on day 0 to 23% on day 9, and 7.7% on day 30 (13).

On the other hand, it was found that Staphylococcus had good susceptibility to cefotaxime (19,25,28), ceftriaxone (10), azithromycin (19), clindamycin (25,26), amoxicillin/clavulanic acid (24,27), ciprofloxacin (25,28), and moxifloxacin (11). Streptococcus presented good susceptibility to ampicillin, ciprofloxacin, gentamicin, ceftazidime (19), ceftriaxone, moxifloxacin (10), amoxicillin/clavulanic-acid (24), linezolid (26), clindamycin (26), and amoxicillin (12,13). Peptostreptococcus spp. showed susceptibility to amoxicillin-clavulanic acid (20,24), imipenem plus cilastatin (20), clindamycin (20), linezolid (26), and metronidazole (23). Gram-negative microorganisms displayed susceptibility to levofloxacin, amoxicillin-clavulanic acid (11), and metronidazole (23,26). Lastly, *Escherichia coli* was sensitive to amikacin (28), ampicillin and ciprofloxacin (19). Klebsiella spp. showed susceptibility to ceftriaxone (27), carbenicillin (27), amikacin (27,28), and imipenem (27). *P. aeruginosa* and *Citrobacter koseri* also were susceptible to ciprofloxacin (19,28), while Enterobacter cloacae had susceptibility to azithromycin (19).

Responding to the third question of this scoping review, it was found that the antimicrobials to which the microorganisms studied presented greater resistance were penicillin (17,18,23-25,28-30), amoxicillin (12,21,26,27,29), erythromycin (24,28,30), and metronidazole (18,21,23,26).

All studies included in this review fully met at least 75% of the defined quality criteria (9), therefore, they were classified as of good quality (Table 2). Nevertheless, it is important to highlight that the studies included in this review presented great heterogeneity in their designs, reflected in the exploration of different classes of antimicrobials, great variability in the characteristics of the patients studied, and variability in the microbiological identification and the microorganisms studied, among other characteristics.

**Table 2:**
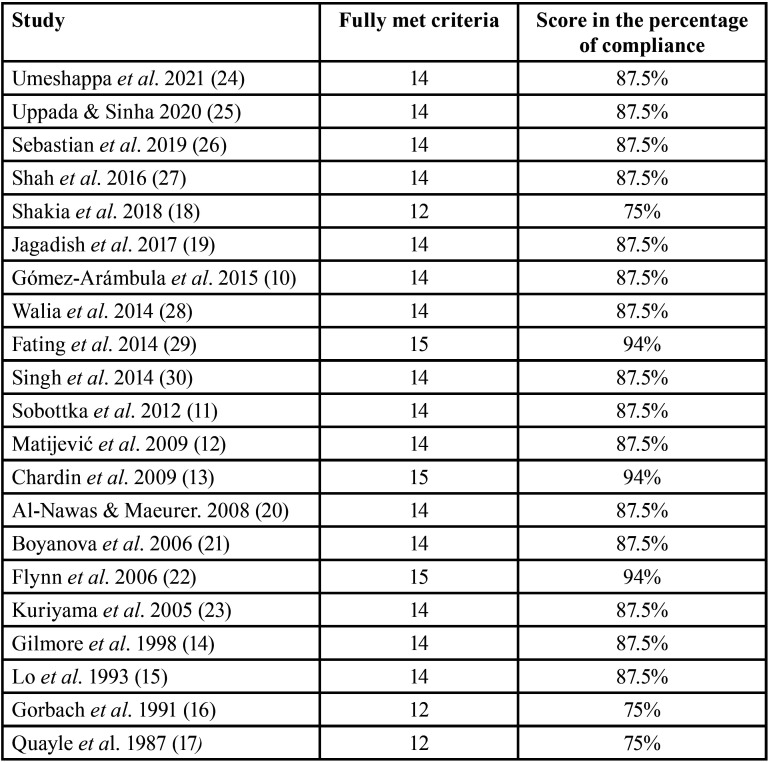
Quality of the selected studies (9).

## Discussion

To the best of the authors’ knowledge, this scoping review is the first to consider the prevalence and proportions of antimicrobial-resistant species in patients with odontogenic infections. Whereas adjunctive antimicrobials are helpful implements in the therapy of some oral cavity infections, latent concerns occur regarding variations in the oral species as a consequence of their management (31,32). These issues involve the three questions proposed in this scoping review.

It is important to note that in this review, only studies containing phenotypic analysis were evaluated, understanding that the manifestation of antibiotic resistance genes does not essentially reveal the antimicrobial resistance of the microorganisms (33). On the other hand, only prospective studies and RCTs were included in this review, considering that retrospective studies are subject to bias including missing data, classification and interpretation bias in clinical records, and inconsistencies in treatment methods, among others (22).

Although incision and drainage is the first treatment option for odontogenic infections, an adequate knowledge of the microorganisms involved in these infections, in addition to their susceptibility to antimicrobials will allow for establishing an adequate therapeutic regimen (1,19). On many occasions after adequate surgical therapy, patients do not improve. One of the relevant reasons is the presence of bacterial resistance and the selection of the inappropriate antimicrobial (24). Unfortunately, while awaiting laboratory results containing information on the identified microorganisms and their antimicrobial susceptibility, clinicians make an empirical selection of antimicrobials (23).

Microbiological samples from odontogenic infections are characterized by being constituted by a complexity of species, which can vary from aerobes and anaerobes to a mixture of aerobes and anaerobes (24). The proportion of these microorganisms varies between studies due to dissimilar techniques and resources implemented. Herein, regarding the prevalence of antimicrobial-resistant species, the most resistant bacteria were Staphylococcus, *Streptococcus spp*., Peptostreptococcus spp., Bacteroides spp., and Prevotella spp. Among aerobic and facultative anaerobe prevailed, *S. aureus* (12,18,19,25,28), *S. viridans* (15,17,26,27), *K. pneumoniae* (12,19,22,27), *S. milleri* (22), Enterococcus spp. (25), *P. aeruginosa* (28), *P. mirabilis* (19), and *S. coagulases-negative* (12). In obligate anaerobes sampled were Peptostreptococcos spp. (12,15,17,18,20,21,23,24,26,30), Bacteroides spp. (12,14,18,21,24,26,28-30), and Prevotella spp. (11,18,20,21,23,26,30). These microorganisms were resistant to different antimicrobials in dissimilar proportions (Table 1- Table 1 cont.-3).

Penicillin is the antimicrobial traditionally used for odontogenic infections. Unfortunately, and due to its widespread use, it has developed the appearance of resistant microorganisms (34). The device comprises beta-lactamase labor that has been validated in anaerobic Gram-negative bacilli. The occurrence of orofacial odontogenic infections including beta-lactamase generating microorganisms fluctuates from 13% to 39% (24). In this regard, it has been informed that the proportion of β-lactam penicillinase resistance in *S. aureus* samples from hospitals and dental clinics observed in 2020 are comparable to methicillin-resistant *S. aureus* percentages described in 2018 (34). Resistance to penicillin has also been frequent in anaerobes caused by the production of beta-lactamase (24).

Amoxicillin has also been one of the antimicrobials that have been prescribed empirically for the management of odontogenic infections (26). Nonetheless, as found in this review, aerobic microorganisms (12,26), anaerobic bacteria (12,21,269, and mixed pathogens (12,26) presented resistance to this antibiotic. On the other hand, amoxicillin/clavulanic acid has shown good efficacy against aerobic and anaerobic microorganisms, showing superiority in activity to amoxicillin alone (11,20,24,27). The supplement with clavulanic acid broadens the spectrum against Staphylococcus spp. and other anaerobes by conceding beta-lactamase resistance (18). It has also been indicated that clindamycin may be an alternative in cases of inefficacy to amoxicillin (35). Clindamycin has good efficacy in aerobic Gram-positive cocci, including *S. aureus*, *Streptococcus spp*., and most anaerobes, counting penicillin-resistant species such as Bacteroides spp., Prevotella spp., and Porphyromonas (20,25,26). Furthermore, the efficacy of clindamycin and amoxicillin/clavulanic acid in odontogenic infections is comparable (36).

As found in this review, it is widely known that metronidazole does not show efficacy against aerobes (18,21,23), but it does against obligate anaerobes (20,23-26). The combination of metronidazole with penicillin has been recommended because it covers the microbial flora of odontogenic infections, compensating for the limited action of penicillin against beta-lactam anaerobes. The combination of amoxicillin/clavulanic acid plus metronidazole has also shown efficacy against strict anaerobes and facultative anaerobes (24).

First and second-generation cephalosporins have presented efficacy against aerobes and anaerobic Gram-positive cocci, corroborating the results of this scoping review (10,12,19,24,27-30). However, their efficacy against anaerobic Gram-negative rods is unpredicTable. In this regard, it has been reported that cefotaxime (third-generation cephalosporin) has demonstrated *in vitro* efficacy against anaerobic bacteria of the mixed flora of odontogenic infections (24), also confirming the results described here (18,19).

Regarding the group of macrolides, it was observed high resistance to erythromycin (21,23,24,26,28,30); however, the efficacy of azithromycin for the treatment of odontogenic infections shows controversial results (15,19,26). While two studies described good efficacy against Staphylococcus spp. (15,19), other research informed high resistance to aerobic bacteria (80%), anaerobic microorganisms (78%), and 70% of mixed pathogens (26). These high values of resistance to macrolides have also been previously referenced (37).

Controversial susceptibility results were also observed in the quinolone group. Ciprofloxacin demonstrated a good efficacy against S. aureus (24,25,28), Staphylococcus spp., and *Streptococcus spp*. (19), Gram-negative microorganisms, *E. coli*, and *P. aeruginosa* (19). One RCT showed that 98% of pathogens (*S. viridans*, Prevotella spp., Neisseria spp., *Streptococcus anginosus*, and other anaerobes) were susceptible to moxifloxacin (11). Similarly, another RCT indicated that anaerobes, Streptococcus, and Staphylococcus spp. showed low resistance to moxifloxacin (10), while one prospective study described that *S. viridans* and *K. pneumoniae* displayed intermediate susceptibility and high resistance to this antimicrobial, respectively (27). As has been described, methodological and geographical differences in research evaluating bacterial resistance may support these results (7,38).

Interestingly, in this review a considerable number of studies found a changing tendency in terms of the preponderance of anaerobic microorganisms over aerobic ones (14,20,21,24,30). Therefore, it has been recommended that prompt identification and careful management of odontogenic infections by surgical drainage and adjunct antimicrobials are essential to avoid the risk of expansion into adjoining fascial spaces (24). Thus, the elevated proportion of anaerobic microorganisms in the current review underlines the relevance of prospective studies in this field.

In short, different investigations recommend that the combination of amoxicillin plus clavulanic acid is the first line of antimicrobial selection, showing efficacy against most microorganisms involved in odontogenic infections (18,20,24,27,29,30). However, more prospective clinical studies and RCTs are required to evaluate antimicrobial resistance in patients with odontogenic infections in different parts of the world. In this regard, a review involving seven reports that assessed 374 patients from diverse nations worldwide, divulged that antimicrobial resistance frequencies varied rendering to the preceding utilization of antimicrobials (39).

The results described by this scoping review may support clinicians and leaders of public health organizations to create important decisions, as well as to obtain a better consciousness of the relevance of the reasonable management of antimicrobials.

## Conclusions

In summary, most of the microorganisms had resistance to diverse groups of antimicrobials. Suitable antimicrobials must be prescribed founded on the microbial samples, culture susceptibility, and clinical progression of the odontogenic infections. Furthermore, it was observed high levels of resistance to antimicrobials that have been used in local and systemic therapy of oral cavity infections. An issue of concern is the preponderance of anaerobic microorganisms over aerobic ones.
